# Supplementary material for: Polyketide synthase gene clusters in the ericoid mycorrhizal fungus Oidiodendron maius: genome-wide prediction, expression profiling, and functional characterization of knockout mutants
Source: Front Plant Sci. 2026 Sep 16;17:1924430. doi: 10.3389/fpls.2026.1924430 (PMC13623903; doi:10.3389/fpls.2026.1924430)
Supplement: Supplementary file 1 [file SupplementaryFile1.zip › Supplementary Materials/Supplementary Folder 1.zip!/Oidma1_AssemblyScaffolds_Repeatmasked/knownclusterblast/cluster23/jgi.p_Oidma1_200957_mibig_hits.html]

| MIBiG Protein | Description | MIBiG Cluster | MiBiG Product | % ID | % Coverage | BLAST Score | E-value |
| --- | --- | --- | --- | --- | --- | --- | --- |
| AAO34671.1 | methyltransferase | BGC0001277 | Nivalenol\_/\_deoxynivalenol\_/\_3-acetyldeoxynivalenol\_/ | 36 | 88.1502890173 | 200 | 4.3e-51 |
| AAO27746.2 | putative\_methyltransferase | BGC0001278 | Nivalenol\_/\_deoxynivalenol\_/\_3-acetyldeoxynivalenol\_/ | 35 | 88.1502890173 | 198 | 1.7e-50 |
